# Raising the bar for causal inference: PLOS Medicine adopts the TARGET guidelines for target trial emulation studies

**DOI:** 10.1371/journal.pmed.1004796

**Published:** 2025-10-31

**Authors:** Helen Lumbard, on behalf of the PLOS Medicine Staff Editors

**Affiliations:** 1 Public Library of Science, Cambridge, United Kingdom

## Abstract

The TARGET guidelines aim to improve transparency and consistency in reporting of observational studies that emulate target trials. This Editorial announces that PLOS Medicine fully endorses the TARGET guidelines and asks that new submissions of target trial emulation studies adhere to them.

## Teaser

The TARGET guidelines aim to improve transparency and consistency in reporting of observational studies that emulate target trials. In alignment with these goals, *PLOS Medicine* fully endorses the TARGET guidelines and now asks that new submissions of target trial emulation studies adhere to them.

In recent years, the methodological paradigm of target trial emulation (TTE)—the explicit design and analysis of observational data to mimic a hypothetical randomized trial (the “target trial”)—has emerged as a powerful and increasingly common approach for drawing causal inferences when randomized trials are unfeasible or unavailable. Yet, despite its growing uptake in epidemiology, health policy, pharmacoepidemiology, and outcomes research, reporting practices for such studies remain inconsistent and/or incomplete [1]. To address this gap, the international TrAnsparent ReportinG of studies Emulating a Target trial (TARGET) guideline was developed via consensus to define a minimum reporting checklist for observational studies that emulate target trials [2,3].

In recognition of the increasing role of TTEs in medical research, and to promote clarity, reproducibility, and critical appraisal of submitted work, *PLOS Medicine* is announcing that all manuscripts submitted to the journal that rely on TTE (or claim to do so) must adhere to the TARGET reporting guideline as a submission requirement (in addition to any other applicable guidelines such as STROBE and CONSORT, etc.). In practice, authors will be asked to submit a completed TARGET checklist, clearly mapping each checklist item to the relevant manuscript section or supplementary material, and to embed in their manuscript (or supplementary files) explicit exposition of the target trial protocol and how it is emulated in the observational data.

## Why this move is timely and needed

Reviews conducted during the development of the TARGET guidelines found that many TTE studies failed to report essential elements, including the causal contrast (estimand), the precise definition of time zero, eligibility criteria, and sensitivity analyses addressing key assumptions [2]. Without clear reporting, readers and reviewers must often reverse-engineer the underlying trial design, making it difficult to assess bias, evaluate assumptions, or judge the validity of the findings.

Part of the problem is that existing observational research guidelines, such as STROBE and its extensions, do not fully address the specific needs of TTE studies. They do not require a complete specification of the target trial protocol—including population, interventions, assignment strategy, follow-up period, and estimand—nor a transparent mapping of these protocol elements to the observational data. TARGET was developed to fill this gap, providing a checklist of 21 essential items across all manuscript sections, tailored specifically to the demands of TTE research [3,4].

Adopting TARGET improves transparency and strengthens the interpretability of TTE studies. Standardized reporting helps reviewers and readers assess the validity of the design, identify unacknowledged biases, and compare findings across studies. It also encourages authors to describe their methodological choices rigorously, raising overall research quality. Ultimately, clearer reporting supports the uptake of results into practice, policy, and guideline development, enhancing both the credibility and the impact of observational causal research.

## What authors should expect, and how submissions will change

- Checklist requirement*:* Authors of manuscripts employing TTE approaches will be asked, at initial submission, to provide a completed TARGET checklist, with page/line references indicating where each item is addressed (or, if not applicable, why omitted). This transparency aids editors and reviewers in evaluating the completeness of reporting.
- Explicit target trial protocol section: All submissions must include a clearly specified hypothetical target trial protocol: Eligibility criteria, intervention strategies, treatment assignment, follow-up period, outcome definitions, causal contrast(s) (estimands), assumptions, and analysis plan. The manuscript should describe how each of those components is emulated using the observational data (including mapping decisions or limitations). The mapping should include discussion of unmeasured variables, timing decisions (e.g., “time zero”), potential selection or measurement biases, and sensitivity or robustness of analyses.
- Flexibility in presentation, but clarity required: We do not mandate a rigid layout. The ordering of presentation, use of tables or diagrams (e.g., a “protocol → emulation” mapping table), or placement in main or supplementary text is left to authors’ discretion, so long as the information is clearly presented and accessible.
- Peer review and editorial expectations: Reviewers will be asked to judge not only scientific merit, but also compliance with TARGET items (and, where relevant, other guidelines). Manuscripts missing essential elements will receive editorial requests for revision or, in some cases, may be declined on grounds of inadequate transparency.
- Implementation timeline: To give the research community time to adapt, we plan a transition period of 6 months from the date of this editorial, during which manuscripts without full TARGET compliance will be allowed but flagged and encouraged to revise. After that transition window, full compliance will become mandatory for TTE-based submissions.

## Supporting authors through the transition

To support the research community during this transition, *PLOS Medicine* will update its website to link directly to the official TARGET site, which includes resources, guidelines, and the full checklist. We encourage authors to consult the TARGET website and templates to help structure their reporting. Throughout the transition period, we welcome feedback from the community and will remain open to reasonable accommodations or clarifications for edge cases.

It is important to emphasize that adopting TARGET is not a prescriptive mandate for methodology; it is a reporting standard. Authors are free to choose or develop novel methods and modeling strategies, provided that their design, assumptions, and mapping decisions are transparently described. TARGET is intended to improve clarity and reproducibility, not to serve as a quality score or risk-of-bias assessment, and it should not be treated as such.

## A call to the research community

The widespread adoption of transparent and rigorous reporting standards is a hallmark of scientific maturity. In randomized trials, the CONSORT statement transformed how trials are reported, reviewed, and appraised. In observational research, STROBE has served a similar role. As causal inference methods evolve, particularly TTE, reporting practices must continue to advance to match these innovations.

We encourage authors, methodologists, and readers to engage with the TARGET initiative, consider the guidance it provides, and apply the guidelines in their reporting. In doing so, we can help ensure that TTE studies are conducted and communicated in a transparent, clear, and reliable manner.

## Abbreviations

**:**
TARGET: TrAnsparent ReportinG of studies Emulating a Target trial
TTE: target trial emulation

## References

[1] Hansford HJ, Cashin AG, Jones MD, Swanson SA, Islam N, Douglas SRG, et al. Reporting of observational studies explicitly aiming to emulate randomized trials: a systematic review. JAMA Netw Open. 2023;6(9):e2336023. doi: 10.1001/jamanetworkopen.2023.36023 37755828 PMC10534275

[2] Hansford HJ, Cashin AG, Jones MD, Swanson SA, Islam N, Dahabreh IJ, et al. Development of the TrAnsparent ReportinG of observational studies Emulating a Target trial (TARGET) guideline. BMJ Open. 2023;13(9):e074626. doi: 10.1136/bmjopen-2023-074626 PMC10503363 37699620

[3] Cashin AG, Hansford HJ, Hernán MA, Swanson SA, Lee H, Jones MD, et al. Transparent reporting of observational studies emulating a target trial-the TARGET statement. JAMA. 2025;334(12):1084–93. doi: 10.1001/jama.2025.13350 40899949 PMC13084563

[4] Hansford HJ, McAuley JH, Cashin AG. Improving reporting of observational studies of interventions: the TARGET guideline. PLoS Med. 2025;22(10):e1004787. doi: 10.1371/journal.pmed.1004787 41091811 PMC12543274

